# Impact of using bitewing radiographs alone or in combination with clinical information on treatment decisions

**DOI:** 10.1590/0103-644020246005

**Published:** 2024-12-16

**Authors:** Gabriele Soares Maydana, Vitor Henrique Digmayer Romero, Cacia Signori, Juliana Lays Stolfo Uehara, Françoise Hélène van de Sande, Maximiliano Sérgio Cenci, Anelise Fernandes Montagner, CaCIA collaborative group

**Affiliations:** 1 Dental School, Federal University of Pelotas, Pelotas, Brazil.; 2 Graduate Program in Dentistry, Dental School, Federal University of Pelotas, Pelotas, Brazil.; 3 Radboud University Nijmegen Medical Centre, Nijmegen, The Netherlands.; 4 Uniavan University Center, Balneário Camboriú, Brazil.

**Keywords:** secondary caries, detection, decision-making. radiography, restoration

## Abstract

The combination of different methods has been advocated to increase sensitivity in detecting secondary caries lesions. This cross-sectional study compared the detection of caries lesions around posterior restorations and treatment decisions using bitewing radiographs alone or in combination with clinical information from patient records. The radiographs (n = 212) were randomly distributed into two sequences for assessment across two phases, with a wash-out period of two weeks. In the first phase (X-ray group), the radiographic images were evaluated without clinical information; in the second phase (X-ray/CARS group), the radiographic images were assessed in conjunction with the CARS score (Caries Associated with Restorations or Sealants) and lesion activity. A radiographic classification system for carious lesions around restorations was adapted to classify the bitewing radiographs included in this study. Evaluations were conducted in consensus by a panel of specialists, focusing on the detection of caries around restorations and subsequent treatment decisions. A chi-squared test was used to compare treatment decisions between the groups, and Cohen’s kappa coefficient was employed to assess the agreement of scores. The results showed a significant difference in the distribution of decisions regarding the need for restorative intervention between the groups (p < 0.001), with the X-ray/CARS group leading to more decisions favoring restorative intervention. There was a moderate to good agreement of scores (κ = 0.749). The combination of clinical and radiographic information was found to influence the treatment decision-making process by increasing the likelihood of opting for restorative intervention.

## Resumo

A combinação de diferentes métodos tem sido defendida para aumentar a sensibilidade no processo de detecção de lesões de cárie ao redor de restaurações. Este estudo transversal comparou a detecção de lesões de cárie ao redor de restaurações em dentes posteriores e a decisão de tratamento usando radiografias interproximais isoladamente ou em combinação com as informações clínicas. As radiografias (n = 212) foram organizadas em 2 sequências aleatórias para avaliação em 2 fases, com um intervalo de 2 semanas. Na primeira fase (grupo RX), as imagens radiográficas foram avaliadas sem informações da avaliação clínica; e na segunda fase (grupo RX/CARS), as imagens radiográficas foram avaliadas associadas ao escore CARS (Caries Associated with Restorations or Sealants) e à atividade da lesão. Um sistema de classificação radiográfica para lesões de cárie ao redor de restaurações foi adaptado para classificar as radiografias interproximais incluídas neste estudo. As avaliações foram realizadas em consenso por um painel de especialistas, considerando a detecção de cárie ao redor da restauração e decisão de tratamento. Um teste Qui-quadrado foi realizado para comparar as decisões de tratamento entre os grupos, e um kappa de Cohen para avaliar a concordância dos escores. Os resultados mostraram uma diferença significativa na distribuição de decisões de necessidade de intervenção restauradora entre os grupos (p < 0,001), com o grupo RX/CARS resultando em mais decisões de intervenção restauradora, e uma concordância moderada a boa dos escores (κ = 0,749). A combinação de informações clínicas e radiográficas influencia o processo de tomada de decisão de tratamento, aumentando a probabilidade de intervenção restauradora.

## Introduction

Secondary caries, or caries lesion around restorations, have been identified as the leading cause of restoration failure and the need for intervention ^1^ ^,^ ^2^ ^,^ ^3^ ^,^ ^4^. The decision to replace restorations due to caries is a routine task for dentists worldwide ^5^. Diagnosing secondary caries presents a significant clinical challenge, particularly when the lesion is located in the interproximal region and associated with resin composite restorations, as factors such as marginal staining and defects can confound clinicians ^6^. The combination of visual-tactile assessment (which allows confirmation of findings) with bitewing radiography (which enables the detection of clinically inaccessible lesions) has been established as a standard for caries detection, especially in interproximal areas ^5^.

However, clinicians often rely heavily on radiographic findings to guide their diagnostic decisions. Sole reliance on radiographs for assessing dental restorations can lead to erroneous decisions, as radiographs are not always accurate in predicting the presence or absence of cavities, and the detection of marginal gaps may result in false-positive and false-negative diagnoses ^3^. Additionally, the presence of adhesive liners under restorations and dental materials with low radiopacity can be misinterpreted as secondary caries ^7^. Radiographic interpretation remains challenging for dentists, contributing to variability in diagnosis and treatment decisions ^8^ ^,^ ^9^, and these factors may lead to unnecessary interventions ^7^.

Does the radiographic examination add value? Indiscriminate use of radiography for caries detection may result in overdetection, unnecessary patient exposure to radiation, and increased healthcare costs ^10^. Therefore, it is crucial to prioritize measures that prevent false-positive diagnoses, minimize healthcare expenses, and avoid overtreatment ^11^. Advanced image-based artificial intelligence diagnostic systems are emerging ^12^ ^,^ ^13^ ^,^ ^14^, making it necessary to understand the role of radiographic images in diagnosing secondary caries and in subsequent treatment decisions. This study aimed to compare the detection of caries lesions around posterior restorations and the treatment decisions using bitewing radiographs alone or in combination with clinical information about the restoration. The study hypothesis was that diagnosis and treatment decisions would be consistent whether radiographic results were analyzed independently or alongside clinical information.

## Materials and Methods

### Study Design

The protocol of this cross-sectional study was published on the Open Science Framework platform (DOI: 10.17605/OSF.IO/TX3ZG). This cross-sectional study was nested in a randomized clinical trial, named Caries Cognition and Identification in Adults (CaCIA) ^15^. The clinical trial is registered on the clinicaltrials.gov platform (NCT03108586) and was approved by the Local Research Ethics Committee (n° 1.625.236/2016).

The research question was: the detection of secondary caries lesions and the suggestion for treatment changes if combining clinical data and radiographic images? For this, bitewing radiographs of posterior permanent teeth were analyzed from patient records. Good quality radiographs of posterior restorations, involving or not the proximal surface, were selected. The selected radiographs were evaluated in two phases by a panel of experts. In phase 1, the panel members assessed only the radiographic images without clinical information (X-ray group). In phase 2, a new sequence of the same radiographic images was evaluated, associated with the clinical information - CARS (Associated with Restorations or Sealants) score and lesion activity ^16^ (X-ray/CARS group). The panel of experts had a washout period of 2-week between each assessment phase. The primary outcome was the treatment decision (restorative intervention or no intervention).

### Sample

Conventional bitewing radiographs were obtained from patient records of the randomized clinical trial CaCIA ^15^, which investigated the impact of using different visual criteria for assessing caries lesions around restorations in patients aged between 18 and 60.

To be included in the study, patients’ records should present:

1. Data from restoration clinical assessments (CARS score; lesion activity) performed by a trained examiner using visual inspection.
2. Good quality bitewing radiographs of vital and non-vital posterior teeth with amalgam or resin composite restorations, involving proximal and/or occlusal surfaces.

Cases without appropriate radiographs due to poor image quality were excluded. In total, 212 bitewing radiographs were included in the sample.

For sample preparation, the conventional bitewing radiographs were photographed. Subsequently, each digital image was processed with a black and white filter using photo editing software (Adobe Photoshop CS6, Adobe Systems Incorporated, USA), to standardize them. These images were inserted into a PowerPoint file (PowerPoint, Microsoft, USA) with a black background for analysis by the panel. The radiographs were identified with the case number, tooth number, and the surfaces that needed to be evaluated by the panel.

A researcher not involved in the radiograph assessment performed sample collection, preparation, and randomization of the cases. Two randomization sequences were generated, one for phase 1 (radiography alone - X-ray group) and another for phase 2 (radiography associated with CARS criteria and caries activity status - X-ray/CARS group).

### Radiographic scoring system

**Figure 1:**
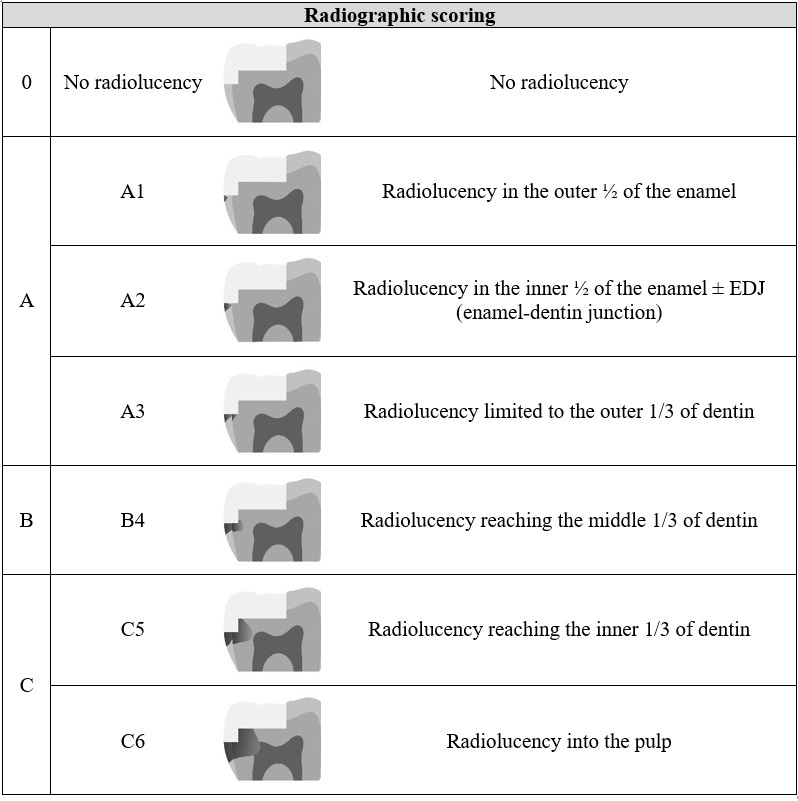
Radiographic scores for classifying caries around restorations.

Based on the radiographic classification of primary caries of the "ICDAS Radiographic scoring system," available in the "International Caries Classification and Management System - ICCMS™ Guide for Practitioners and Educators" ^17^, a radiographic scoring system for caries lesions around restorations was created to classify the bitewing radiographs included in this study. The system was organized into levels (0, A, B, and C) and sublevels (RA1, RA2, RA3, RB4, RC5, RC6). Restorations that did not show radiolucency around them were classified as score 0. Score A was considered when it was possible to visualize radiolucency only up to the outer third of dentin, with RA1 restricted to the outer half of enamel, RA2 up to the inner half of enamel, and RA3 with radiolucency up to the outer third of dentin. Score B (sublevel RB4) was considered when radiolucency reached the middle third of dentin. Score C was considered when it was possible to visualize radiolucency up to the pulp, with C5 when radiolucency reached the inner third of dentin and C6 when radiolucency reached the pulp. Figure 1 presents the developed radiographic scoring system.

The radiographic scores for classifying caries lesions around restorations are described in Figure 1. The complete scoring, with level and sublevels (0, A1, A2, A3, B4, C5, and C6) was created to promote a complete radiographic scoring system. However, in this study, the radiographs were classified based on the score using only levels 0, A, B, or C.

### Radiographic assessment

The assessments of digital radiography images were conducted on a 13-inch computer screen. The radiographs were analyzed considering the radiographic level score based on the presence and extent of radiolucency compatible with carious lesions around the restoration (0, A, B, or C) (Figure 1). When radiolucency was incompatible with caries lesions around restorations, such as those caused by an adhesive layer, lining material, bubbles in the restoration, or residual caries, the radiographic score was classified as 0. For the decision-making (need for restorative intervention), the experts could use the score 0 for 'no intervention', 1 for 'more information is required', and 2 for 'intervention'. Radiographically, the restorations were evaluated in their entirety and were classified according to the face that presented the greatest radiolucency.

The sublevels (A1, A2, A3, B4, C5, and C6) were not used in the panel assessments, as the intervention indication does not vary within the same level.

### Panel of Experts

The panel of experts consisted of four dentists (VHDR, JLSU, CS, and AFM) with experience in cariology and restorative dentistry. Before the assessments, the panel experts underwent theoretical and practical training, with case discussions (not included in this study).

### Assessment phases

**Figure 2:**
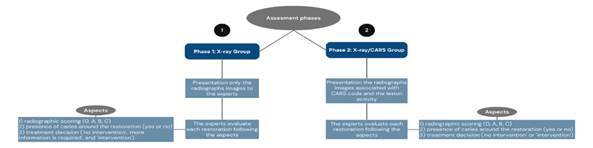
Flowchart of the study's assessment phases.

The study was conducted in two assessment phases. Figure 2 presents the flowchart of the study's assessment phases.

*Phase 1* - Assessment based only on radiographs (X-ray group): Initially, the detection of caries lesions around restorations and decision-making were based solely on radiographs, and the following aspects were evaluated for each restoration:

1. Radiographic scoring (0, A, B, or C)
2. Presence of caries around the restoration (yes or no)
3. Treatment decision ('no intervention', 'more information is required', and 'intervention').

In this phase, scores 0 and A did not indicate the need for restorative intervention ('no intervention' decision), B indicated the need for further information ('more information is required' decision), and C indicated the need for restorative intervention ('intervention' decision). The 'no intervention' decision indicated no restorative intervention was needed. The 'more information is required' decision indicated that the X-ray image did not provide sufficient information. The 'intervention' decision indicated that restorative intervention was necessary.

*Phase 2* - Assessment based on radiographs associated with clinical data (X-ray/CARS group): To evaluate the influence of clinical characteristics on decision-making, the same radiographic images presented in Phase 1 were randomly reordered and presented to the panel, and the same aspects (radiographic scoring, presence of caries around the restoration, and treatment decision) were again evaluated. The radiographs and clinical assessment data previously collected by a trained examiner were presented. The available clinical data for each restoration included the CARS (Caries Associated with Restorations or Sealants) code and lesion activity ^16^. This clinical information was collected during visual inspections in the follow-ups of the CaCIA trial. Each restoration was scored from 0 ('healthy tooth surface with restoration') to 6 ('extensive distinct cavity with visible dentin') according to the CARS criteria (Supplementary material). The panel associating the radiographic information with clinical criteria assessed the treatment decision restorative ‘intervention’ or ‘no intervention’ decision. The panel did not have access to clinical digital images or visual inspections of the restorations. Restorative intervention was recommended if lesions reached the dentin and showed activity, even if the radiographs did not show radiolucency consistent with caries lesions.

In both phases, experts individually assessed each case and after individual assessments, the panel engaged in discussions to reach a consensus on each aspect. If no consensus was reached (50% of cases), it was noted, and the decision considered for analysis by a fifth researcher was the most conservative approach.

The panel of experts had a two-week interval (washout period) between each assessment phase.

### Statistical analysis

A descriptive analysis of absolute and relative frequencies of the included radiograph variables and assigned classifications was conducted. To compare the restorative intervention decisions between the different groups (X-ray group vs -ray/CARS group), the variables were dichotomized, with 'no intervention' and 'more information is required' combined. The decision 'more information is required' was only used in the assessment phase where the radiograph was not associated with any clinical information (X-ray group). A chi-squared test was performed to compare the decisions between the groups. Cohen's kappa and weighted kappa statistics were calculated to assess agreement between the groups' radiographic scores between phases 1 and 2, utilizing quadratic weights for the weighted analysis to reflect the ordinal nature of data. All statistical analyses were performed using Python programming language, version 3.10.12. The code was executed in a Jupyter Notebook environment hosted on Google Colab, a cloud-based Python interpreter. A significance level of 5% was considered in all analyses.

## Results

Descriptive data are presented in Table 1. A total of 212 radiographs of posterior restorations were analyzed in this study. Proximal involvement was observed in 108 cases, while 104 did not show proximal involvement. Categorizing based on the CARS code revealed 125 cases with a score of 0 (59%); 39 with a score of 1 (18%); 31 with a score of 2 (15%); and 17 with a score of 3 (8%). Caries activity was noted in 13 cases, while no activity was observed in most cases (n=199, 93.9%). Radiographic scoring in the X-ray and X-ray/CARS groups was predominantly 0 (89.1%), with 7 (3.3%) and 16 (7.6%) decisions for intervention, respectively.

Cohen's kappa statistic was applied to evaluate the level of agreement between the radiographic scoring between phases 1 and 2, yielding a score of 0.707, indicating moderate agreement. Further analysis utilizing a weighted kappa statistic, with quadratic weights to account for ordinal data, resulted in a higher agreement score of 0.749.

In assessing the treatment decisions across the two groups (X-ray and X-ray/CARS) it was found that a large majority, specifically 195 (92.4%) cases, had no restorative intervention recommended in both groups (Table 2). Moreover, 6 (2.8%) cases had a restorative intervention recommended in both the X-ray and X-ray/CARS groups. Only one case had a restorative intervention suggested in the X-ray group but not in the X-ray/CARS group. Moreover, in 10 (4.2%) cases a restorative intervention was recommended only when assessed with X-ray/CARS. The Chi-square test showed a significant difference between the decisions (p<0.001), with the X-ray/CARS group resulting in more restorative intervention decisions. A cross-table of the radiographic scoring for X-Ray and X-Ray/CARS groups is presented in the Supplementary material (Table S1).

**Table 1:** Absolute and relative descriptive results of the included radiographs (n = 212)

Variables	N (%)
Type of teeth	
Premolar	49 (23.1)
Molar	163 (76.9)
Proximal restoration	
No	104 (49)
Yes	108 (51)
CARS CODE Cat	
0	125 (58.9)
1	39 (18.4)
2	31 (14.6)
3	17 (8.1)
CARS Activity	
No	199 (93.9)
Yes	13 (6.1)
Radiographic Scoring (X-ray)	
0	176 (83.0)
A	20 (9.4)
B	7 (3.3)
C	9 (4.2)
Radiographic Scoring (X-ray/CARS)	
0	174 (82.0)
A	22 (10.4)
B	8 (3.8)
C	8 (3.8)
Intervention (X-ray)	
General No*	205 (96.7)
No	192 (90.6)
More information	13 (6.1)
Yes	7 (3.3)
Intervention (X-ray/CARS)	
No	197 (92.9)
Yes	15 (7.1)

*General No (combined data from 'no intervention' and 'more information is required')

**Table 2:** Cross-table of restorative intervention decisions between X-ray and X-ray/CARS groups.

	X-Ray: No intervention	X-Ray: Intervention
X-Ray/CARS: No intervention	196 (92.4%)	1 (0.47%)
X-Ray/CARS: Intervention	9 (4.2%)	6 (2.8%)

**Figure 3:**
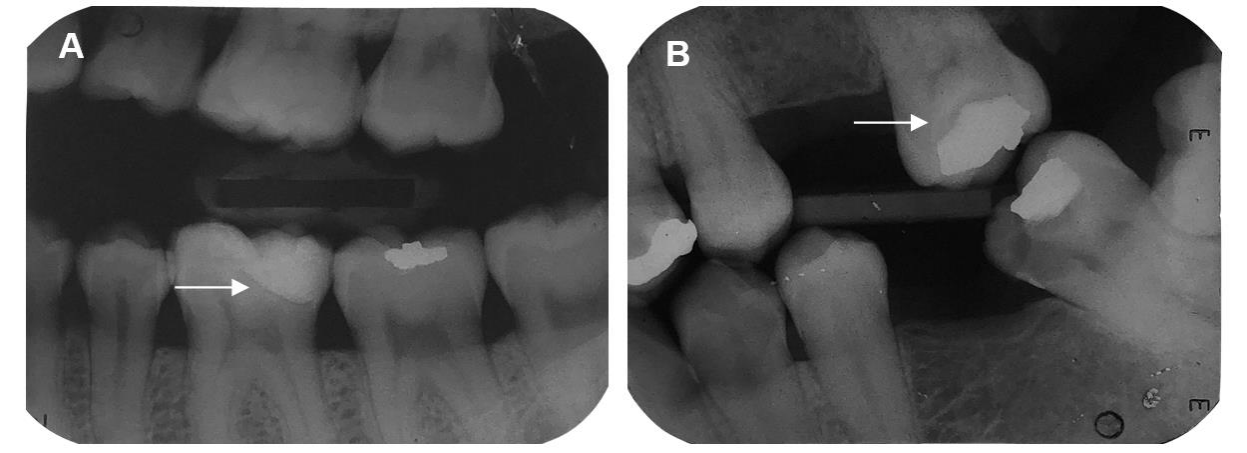
Examples of bitewing radiographs showing the presence of radiolucency incompatible with lesion around restoration. A: White arrow showing radiolucency line around the restoration compatible with an adhesive layer. B: White arrow showing radiolucency below the restoration compatible with residual caries issue after selective caries removal.

**Figure 4:**
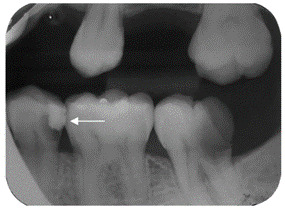
The radiographic aspect scored as C for the presence of a lesion around the restoration that clinically presented CARS 0 and activity 0, possibly due to difficulty in visualization during the clinical examination.

Some radiographs included in this study showed radiolucency associated with the tooth-restoration interface incompatible with a secondary caries lesion (Figure 3). Additionally, there was a case where the secondary caries lesion was detected only by the radiography (Figure 4).

## Discussion

This study assessed the influence of incorporating bitewing radiographs on the detection of caries lesions around restorations and its impact on the decision-making process, comparing the use of bitewing radiographs alone versus in combination with clinical information from patient records. The findings showed a disparity between utilizing bitewing radiographs alone and their combination with clinical data for identifying caries lesions around dental restorations, subsequently affecting treatment decisions, with an increase in restorative intervention decisions, thereby rejecting the study hypothesis.

The combination of detection methods, incorporating both radiographic images and clinical information (CARS), resulted in a higher frequency of restorative treatment decisions. This finding suggests that exclusive reliance on isolated radiographic examinations may be limited when making treatment decisions, highlighting the crucial role of clinical information in facilitating well-informed decision-making. Clinical information is essential for informed decision-making ^18^. While more non-intervention decisions were noted in the X-ray group, further information was requested in 6% of cases to determine the need for intervention. However, when radiographic examination was combined with clinical criteria, the necessary information for deciding whether to intervene became readily accessible.

Radiographic classification systems can aid in detecting caries lesions ^9^. Inspired by the radiographic classification of primary caries lesions described in the ICCMS ^17^, the radiographs in this study were evaluated using a proposed radiographic score for caries lesions around restorations, categorized into levels A, B, or C (Figure 1). The findings indicated moderate agreement in using this system. Scores 0 and A indicated no need for intervention, B ranged from requiring further information to indicating intervention, and C suggested the need for restorative intervention. In the present study, most radiographs were classified as score 0 (89.1%), resulting in no indication of restorative intervention. In radiographic evaluations, secondary caries is commonly defined as the presence of radiolucency associated with a restoration ^19^. However, not all radiolucency at the tooth-restoration interface on radiographs corresponds to a secondary caries lesion ^19^ ^,^ ^20^. Caution is required during radiographic interpretation, as radiolucency may indicate a thick adhesive layer, residual caries tissue after selective caries removal (Figure 3), or even be masked by certain restorative materials. Therefore, it is crucial to consider these differential aspects during diagnosis and subsequent decision-making. The radiolucency observed in the radiographs in this study was classified using the proposed radiographic scores for caries around restorations only in cases where a secondary caries lesion was likely. The results showed no statistical difference between the evaluation of radiographic scores across different groups, indicating the reproducibility of the scoring system used.

Studies have reported that visual criteria used in the clinical evaluation of restorations can directly influence treatment decisions regarding whether to intervene ^21^ ^,^ ^22^. In the present study, clinical information from patient records was based on the CARS visual criteria and lesion activity, which were considered by the panelists during the decision-making process. This is why the X-ray/CARS group resulted in more restorative intervention decisions, as lesion activity was noted as a decisive factor streamlining the decision-making process regarding the need for intervention.

Diagnosing caries lesions must consider lesion activity. Among the available criteria, CARS is an excellent alternative for evaluating caries lesions around restorations, as it considers lesion activity ^22^. Research findings demonstrate that lesion activity has a substantial impact and can lead to more assertive treatment decisions in both initial and advanced lesion stages ^23^ ^,^ ^24^. In the X-ray/CARS assessment, restorative interventions were recommended when lesions showed both activity and extension into dentin, even in cases where radiographic evaluation did not reveal radiolucency indicative of a carious lesion around the restoration. In cases of discrepancies between radiography and clinical information, clinical information remains imperative, and factors such as activity, lesion extension, consistency, and access to hygiene may be relevant for informed decision-making ^25^.

This study showed that radiographic assessment alone should be avoided; however, when appropriately indicated and combined with clinical information, it aids in decision-making, particularly in cases where clinical evaluation is challenging. The indiscriminate use of radiography for caries detection and improper indication can lead to unnecessary patient exposure to radiation and increased healthcare costs ^10^. Avoiding false-positive detection and overtreatment is a priority, making it imperative to implement measures to minimize these outcomes, thereby reducing healthcare costs ^11^. The prevalence of secondary caries is not well-defined, complicating an accurate estimate of its impact on healthcare costs ^10^. However, evidence points to a considerable impact, as the replacement or repair of posterior restorations diagnosed with secondary caries accounts for a significant portion of operative treatments performed in clinical practice ^26^.

Proximal involvement was observed in half of the sample and was positively associated with the need for intervention, making such cases approximately 4.6 times more likely to require treatment. Caries lesions around restorations typically occur in areas where biofilm accumulates, often affecting the cervical margins of proximal restorations ^27^, posing a challenge for clinicians in lesion detection. Strictly occlusal restorations and the occlusal portion of occlusal-proximal restorations are rarely affected by caries, as they are less conducive to dental biofilm stagnation. However, secondary lesions on the occlusal surface can be readily visualized during visual-tactile assessment ^28^. In this study, it was observed that bitewing radiographs are limited in detecting occlusal lesions around restorations. Radiographic images are two-dimensional, and overlapping structures may obscure the lesion ^29^. In such cases, clinical characteristics from visual and tactile inspection are more decisive for lesion management, while the radiographic appearance may be a confounding factor. Radiographic detection is highly accurate for cavitated proximal lesions ^30^ but is limited for occlusal lesions.

It is important to highlight that bitewing radiographic evaluation is a complementary tool to clinical assessment and is often essential for diagnosing secondary caries, particularly at the cervical level. However, information about the presence or absence of a cavity and lesion activity must be confirmed through clinical examination ^29^. In a cost-effectiveness study on proximal secondary caries detection methods, radiographic detection verified by tactile assessment was less expensive but had limited effectiveness, indicating that secondary lesion detection methods should be combined ^31^. Radiographic assessment becomes particularly valuable in cases such as lesions on the gingival margin of proximal restorations, which are difficult to access through visual assessment alone (Figure 4).

A limitation of this study is the low number of events (caries lesions around restorations) in the sample, as the majority (89%) of restorations were radiographically classified as score 0 (no radiolucency), and only 17% presented a diagnosis of secondary caries lesions involving dentin. Another limitation is that the data were not compared with decisions made in clinical trials based on the criteria, and it was not verified whether caries lesions were indeed present upon intervention. Moreover, the radiographs were classified using levels (A, B, or C), and sublevels (A1, A2, A3, B4, C5, and C6) were not used in the panel assessments since the indication for restorative intervention does not vary within the same level.

Despite these limitations, there are strengths to be highlighted. The assessments were conducted by a panel of specialists through consensus discussions for each case, demonstrating the impact of incorporating bitewing radiographs on detecting caries lesions around restorations and its effect on treatment decisions. The study also identified significant predictors for the need for intervention, including proximal involvement, caries activity, and advanced CARS code lesions.

## Conclusion

The combination of clinical and radiographic information influences the treatment decision-making process by enhancing the likelihood of opting for restorative intervention.

## Appendix

**Supplementary Table:** S1. Cross-table of radiographic scoring between X-Ray and X-Ray/CARS groups.

	X-Ray/CARS: 0	X-Ray/CARS: A	X-Ray/CARS: B	X-Ray/CARS: C
X-Ray: 0	167 (96.5)	6 (3.4)	2 (1.1)	1 (0.5)
X-Ray: A	3 (15.0)	15 (75.0)	2 (10.0)	0 (0.0)
X-Ray: B	2 (28.6)	1 (14.3)	4 (57.1)	0 (0.0)
X-Ray: C	2 (22.2)	0 (0.0)	0 (0.0)	7 (77.8)
